# High TSH levels during TSH suppression therapy in DTC postoperative patients are associated with low DIO2 expression in the thyroid and impaired thyroid hormone sensitivity

**DOI:** 10.3389/fendo.2025.1607927

**Published:** 2025-10-01

**Authors:** Jialu Wu, Juan Huang, Zhe Yan, Anqi Yuan, Yifei Song, Hui Huang

**Affiliations:** ^1^ Department of Endocrinology and Metabolism, West China Hospital, Sichuan University, Chengdu, China; ^2^ Department of Basic Courses, Chongqing Medical and Pharmaceutical College, Chongqing, China

**Keywords:** thyroid papillary carcinoma, levothyroxine, TSH suppression therapy, type 2 deiodinase, thyroid hormone sensitivity indices

## Abstract

**Introduction:**

Some patients with differentiated thyroid cancer (DTC) exhibit persistently elevated TSH levels despite undergoing TSH suppression therapy after total thyroidectomy. This study aims to investigate the expression of type 2 deiodinase (DIO2) in thyroid tissues, as well as central and peripheral thyroid hormone sensitivity in these patients.

**Methods:**

A total of 162 DTC patients who underwent total thyroidectomy and received TSH suppression therapy were enrolled. Patients were stratified into Low TSH and High TSH groups based on postoperative TSH levels. Thyroid function and thyroid hormone sensitivity indices were compared before and after treatment, and DIO2 expression in normal thyroid tissue was analyzed.

**Results:**

All patients showed significantly elevated FT4 levels after TSH suppression therapy. Postoperative FT3 levels in the High TSH group were lower than preoperative levels. Notably, the High TSH group exhibited significantly lower FT3/FT4 ratios and higher central thyroid sensitivity indices than the Low TSH group. Immunohistochemistry revealed reduced DIO2 expression in thyroid tissues of the High TSH group. This significant difference persisted when analysis was restricted to patients with normal FT4 levels in both groups.

**Discussion:**

DTC patients with persistently high postoperative TSH levels display lower DIO2 expression in normal thyroid tissues. These findings suggest that poor responders to TSH suppression therapy inherently exhibit impaired central and peripheral thyroid hormone sensitivity, which may be exacerbated by total thyroidectomy. TSH index shows predictive value for L-T4 monotherapy efficacy. These results highlight the need for personalized TSH suppression strategies in DTC management.

## 1 Introduction

Thyroid cancer is the most common malignant tumor of the endocrine system (1). Differentiated thyroid cancer (DTC) accounts for more than 90% of all thyroid cancers. Presently, the recommended treatments for DTC include surgery, thyroid selective radioactive iodine-131 treatment and thyroid stimulating hormone (TSH) suppression therapy. TSH is essential for the proliferation of both normal thyroid cells and thyroid cancer cells. In DTC cells that express functionally active TSH receptors, TSH promotes growth by activating the cyclic adenosine monophosphate signaling pathway (2–4). TSH suppression therapy utilizes levothyroxine (L-T4) to maintain subnormal TSH levels, thereby inhibiting thyroid cancer cell proliferation and suppressing tumor growth (5).

Previous studies have shown that in patients receiving TSH suppression therapy, some showed significantly lower free triiodothyronine (FT3) levels when TSH was reduced to the target range (6, 7). Some scholars believed that these patients need to undergo combination therapy consisting of thyroxine (T4) and triiodothyronine (T3) rather than monotherapy with L-T4 (8, 9). However, the mechanism underlying the postoperative decline in FT3 levels remains unclear. In addition, for some patients, even when free thyroxine (FT4) reaches or exceeds the upper limit of the reference range, the TSH still fails to normalize, not mention reaching the target value. This puts clinicians in a dilemma when formulating medication regimens, as they are caught between the goal of achieving the TSH target and the risk of medication-induced hyperthyroidism (10).

Thyroid sensitivity indices, including thyroid-stimulating hormone index (TSHI), thyrotroph thyroxine resistance index (TT4RI) and FT3/FT4 ratio, are used to assess the responsiveness of central or peripheral tissues to thyroid hormones. Central sensitivity to thyroid hormone was evaluated by TSHI and TT4RI. The higher the values are, the lower the central sensitivity to thyroid hormones is (11). In healthy individuals, the thyroid gland produces all T4 and approximately 15-20% of T3, while the remaining 80-85% of T3 is generated from T4 by the action of type 1 deiodinase (DIO1) and type 2 deiodinase (DIO2) in peripheral tissues (12). The FT3/FT4 ratio is used to evaluate peripheral thyroid hormone sensitivity (13). Higher FT3/FT4 ratio suggests higher peripheral thyroid hormone sensitivity (14).

This study aimed to investigate the mechanisms underlying elevated TSH levels after TSH suppression therapy in patients undergoing total thyroidectomy for DTC and to explore the associations with thyroid DIO2 expression and central and peripheral thyroid hormone sensitivity.

## 2 Materials and methods

### 2.1 Study design

This clinical follow-up research collected data from 2019 to 2023, on patients who underwent total thyroidectomy and received TSH suppression therapy in our hospital. According to the 2015 American Thyroid Association Management Guidelines for Adult Patients with Thyroid Nodules and Differentiated Thyroid Cancer (1), as well as the latest Chinese guidelines for DTC (15), while postoperative TSH targets varied according to patients’ risk stratification, the guidelines recommended TSH supression therapy to maintain TSH levels below 2.0 mU/L. Due to the negative feedback regulation of the hypothalamic-pituitary-thyroid (HPT) axis, supplementation with exogenous thyroid hormone often leads to a decrease in TSH levels as FT4 levels increase. We included patients who had normal thyroid function prior to total thyroidectomy and whose FT4 levels increased (and exceeded the median of the normal reference range) postoperatively.

**Figure 1:**
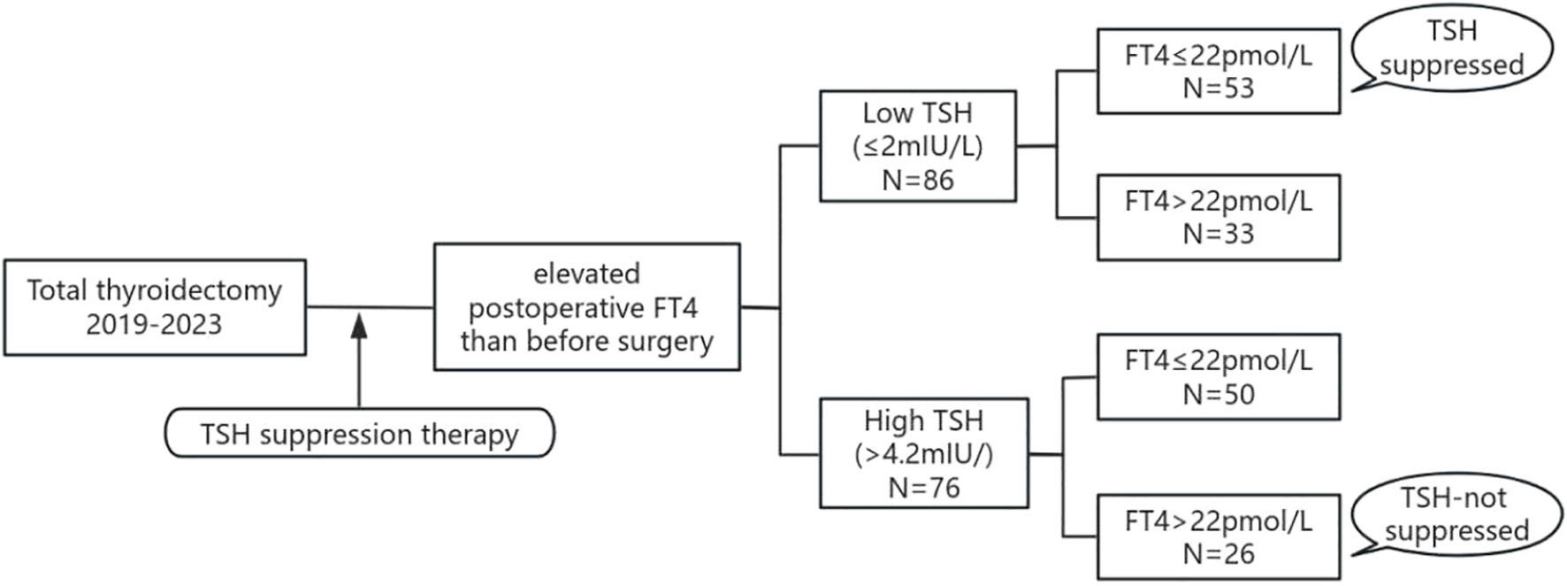
Flow chart of the study population. TSH, thyroid stimulating hormone; FT4, free thyroxine.

Variations exist in both detection methodologies/reagents across institutions and reference ranges among ethnic populations. The reference range of TSH (0.27-4.2 mU/L) and FT4 (12-22pmol/L) have been validated in our population by the Department of Laboratory Medicine of our hospital. In this study, patients with postoperative TSH ≤ 2 mU/L were regarded as meeting the target range and were classified into the Low TSH group (1, 15). Patients were classified to High TSH group when their TSH even can not been suppressed to the normal range. Patients were then categorized into different subgroups based on their FT4 levels: the TSH suppressed subgroup (TSH ≤ 2mU/L and FT4 ≤ 22pmol/L), and the TSH not-suppressed subgroup (TSH > 4.2mU/L and FT4 > 22pmol/L), as shown in **Figure 1**.

The main inclusion criteria were age ≥ 18 years, normal thyroid function maintained preoperatively, normal pituitary functions maintained preoperatively and postoperatively, total thyroidectomy or combined radioactive iodine-131 treatment, pathological diagnosis after surgery confirming differentiated thyroid cancer, postoperative thyroxine suppression therapy, postoperative TSH ≤ 2 mU/L or > 4.2 mU/L, and postoperative FT4 levels were higher than that before surgery.

The main exclusion criteria were as follows: 1) pregnant women, lactating women, and children. 2) Patients with abnormal thyroid function or other endocrine disorders before surgery. 3) Patients with severe or chronic cardiovascular, renal, pulmonary, hepatic, or mental illnesses. 4) Patients taking medications that may affect thyroid hormone metabolism, such as anti-thyroid drugs, corticosteroids, estrogens, amiodarone, beta-blockers, lithium, sucralfate, iron or iodine-containing medications, etc.

### 2.2 Laboratory data

The most recent laboratory data before the total thyroidectomy were recorded. Postoperative follow-up data were collected after at least 6 months of TSH suppression therapy, when thyroid function reached relative stability. The serum levels of TSH, FT3, FT4, Thyroglobulin Antibody (TgAb), and Thyroid Peroxidase Antibody (TPOAb) were measured via electrochemiluminescence using a Roche detection kit and a Roche automatic biochemical analyzer (ModulerEE Electrochemiluminescence E170D) in the Department of Laboratory Medicine of our hospital. The reference ranges for TSH were 0.27-4.20 mU/L, for FT3 were 3.60-7.50 pmol/L, for FT4 were 12.0-22.0 pmol/L. The definitions of positive thyroid autoantibodies were TgAb>115 U/mL and/or TPOAb>34 U/mL. For samples with serum concentrations below or above the assay’s detection limits, the lower or upper limit of detection was assigned and included in statistical analyses.

To evaluate peripheral thyroid hormone sensitivity, the FT3/FT4 ratio was calculated. To evaluate central thyroid hormone sensitivity, TSHI and TT4RI were evaluated. TSHI = ln TSH (mIU/L) + 0.1345 × FT4 (pmol/L) (16). TT4RI = FT4 (pmol/L) × TSH (mIU/L) (16).

### 2.3 Expression of type 2 deiodinase in thyroid tissue

Paraffin-embedded tissue sections were obtained from surgically resected specimens from patients diagnosed with DTC. The adjacent normal thyroid tissues were used to determine the expression of DIO2 by immunohistochemical analysis using a gout polyclonal anti-type 2 deiodinase antibody (NBP1-00178, Novus, USA). Each thyroid section was imaged under 100X and 200X magnification in three random fields of view with a microscope (AX10imagerA2/AX10camHRC, Zeiss, Germany). The quantitative analysis of the average optical density (AOD) was performed with Image J software (https://imagej.net/ImageJ). The mean AOD for each sample was computed based on the mean value of all included slices.

### 2.4 Ethics approval

This study was approved by the Ethics Committee on Biomedical Research, West China Hospital of Sichuan University (No.2022-355). The study was conducted in accordance with the 2013 (7th Edition) Declaration of Helsinki. Informed consent was obtained from all individual participants included in the study.

### 2.5 Statistical analysis

Categorical variables were described as frequencies (percentages). Continuous variables were tested for a normal distribution using the Kolmogorov–Smirnov test. Variables that were not normally distributed were reported as medians (first quartile (Q1) or third quartile (Q3)). Variables with a normal distribution were reported as the means ± standard deviations (SDs). Paired t tests were used for paired samples (normally distributed), while unpaired t tests (normally distributed) or Mann–Whitney U tests (not normally distributed) were used for independent samples. Receiver operating characteristic (ROC) curves were drew and the area under ROC curve (AUC) for each outcome were calculated. A p value < 0.05 was used as a cutoff for statistical significance. All analyses were conducted using SPSS (v23.0; IBM, Armonk, NY, USA) or GraphPad Prism 9.5 (GraphPad, San Diego, CA).

## 3 Results

The baseline characteristics and clinical data of the enrolled participants were presented in **Table 1**. A total of 162 participants, including 85 (52.2%) females with a median age of 40.93 ± 11.74 years old, were included in the data analysis. Among them, 108 (66.7%) patients received radioactive iodine-131 treatment after undergoing total thyroidectomy. Patients had different TNM stage classifications, but none of them had distant tumor metastasis. Considering the patients’ ages, all patients enrolled had stages I~II thyroid cancer according to the TNM Classification of Malignant Tumors (8th edition) (17). Preoperatively, the thyroid function of the patients was within the normal range. Postoperatively, the median dosage of LT4 suppressive therapy used was 1.623 µg/kg. Generally, FT4 levels increased, FT3 levels decreased postoperatively.

**Table 1:** The baseline characteristics and clinical data of enrolled participants.

Variables	N (%)	Variables	Mean ± SD/Median (Q1,Q3)^a^
N	162	Age (yrs)	40.93 ± 11.74
Females	85 (52.5%)	BMI (kg/m^2^)	23.83 ± 3.83
I^131^	108 (66.7%)	preoperative TSH (mU/L)	2.458 ± 0.913
Positive TGAB	23 (14.2%)	preoperative FT3 (pmol/L)	4.986 ± 0.644
Positive TPOAB	18 (11.1%)	preoperative FT4 (pmol/L)	17.139 ± 2.081
T stage		preoperative FT3/FT4 ratio	0.294 ± 0.045
1-2	80 (49.4%)	postoperative TSH	1.023 (0.136,6.415)
3-4	82 (50.6%)	postoperative FT3 (pmol/L)	4.561 ± 0.781
N stage		postoperative FT4 (pmol/L)	22.432 ± 3.122
0	74 (45.7%)	postoperative FT3/FT4 ratio	0.205 ± 0.034
1	88 (54.3%)	L-T4 dosage (ug/kg)	1.623 (1.471,1.786)

^a^:Standard Deviation (SD), first quartile (Q1), third quartile (Q3).

Thyroid function and thyroid hormone sensitivity indices were compared between the Low TSH and High TSH groups (**Table 2**). Prior to total thyroidectomy, there was no significant difference in TSH, FT3/FT4 ratio, TSHI, or TT4RI between the two groups; FT3 and FT4 levels were both higher in the High TSH group. After total thyroidectomy, all patients received L-T4 for TSH suppression therapy. The average dose of L-T4 used in the High TSH group was higher than that in the Low TSH group, but no statistical difference was observed. After sufficient L-T4 treatment, the TSH levels in the High TSH group did not decrease to below the upper limit of the reference range (4.2 mU/L) and were significantly higher than those in the Low TSH group; moreover, patients in the High TSH group had lower FT3 levels, FT4 levels and FT3/FT4 ratios with higher TSHI and TT4RI levels than did those in the low TSH group. Additionally, compared to preoperative levels, the postoperative FT4 levels in both groups were significantly higher; no significant difference was found in FT3 levels (t=-0.354, p=0.724) in the Low TSH group; but the postoperative FT3 levels significantly decreased (t=10.458,p<0.001) in the High TSH group.

**Table 2:** Comparison of thyroid function between the different groups.

Variables		Low TSH (N=86)	High TSH (N=76)	*P*
**Pre-operative**	TSH (mU/L)	2.447 ± 0.863	2.472 ± 0.972	0.863
	FT3 (pmol/L)	4.865 ± 0.640	5.124 ± 0.626	0.010*
	FT4 (pmol/L)	16.737 ± 2.183	17.595 ± 1.870	0.008*
	FT3/FT4 ratio	0.295 ± 0.047	0.294 ± 0.043	0.912
	TSHI	3.079 ± 0.435	3.181 ± 0.499	0.165
	TT4RI	40.530 ± 14.068	43.375 ± 17.832	0.266
**Post-operative**	TSH (mU/L)	0.161 (0.066,0.450)	6.580 (5.018,9.238)	<0.001*
	FT3 (pmol/L)	4.894 ± 0.769	4.179 ± 0.601	<0.001*
	FT4 (pmol/L)	23.318 ± 3.127	21.429 ± 2.814	<0.001*
	FT3/FT4 ratio	0.212 ± 0.033	0.198 ± 0.034	0.009*
	TSHI	1.244 (0.398,2.115)	4.742 (4.463,5.246)	<0.001*
	TT4RI	3.511 (1.580,10.10)	140.1 (109.1,195.2)	<0.001*
L-T4 dosage (ug/kg)		1.561 (1.439,1.786)	1.675 (1.540,1.831)	0.132

*p<0.05.

Thyroid function and thyroid hormone sensitivity indices were further compared between the TSH suppressed subgroup and TSH not-suppressed subgroup (**Table 3**). Compared with the TSH suppressed subgroup, preoperatively, the TSH not-suppressed subgroup had higher levels of FT3, FT4, TSHI and TT4RI; postoperatively, the FT4 was significantly higher but the FT3 was significantly lower, and the mean differences between the two groups were even more pronounced than compared in Low TSH group and High TSH group. Additionally, the FT4 levels in both subgroups were significantly higher compared to preoperative levels; the FT3 levels showed a decrease in TSH suppressed subgroup (t=2.177, p=0.037), with a more significant decline observed in TSH not-suppressed subgroup (t=6.129, p<0.001).

**Table 3:** Comparison of thyroid function and thyroid hormones sensitivity between different sub-groups.

Variables		TSH suppressed (TSH<2 and FT<22)	TSH not-suppressed (TSH>4.2 and FT4>22)	*P*
**Pre-operative**	TSH (mU/L)	2.325 ± 0.894	2.531 ± 0.959	0.398
	FT3 (pmol/L)	4.882 ± 0.624	5.286 ± 0.588	0.014*
	FT4 (pmol/L)	15.959 ± 1.530	18.456 ± 1.885	<0.001*
	FT3/FT4 ratio	0.308 ± 0.046	0.288 ± 0.036	0.075
	TSHI	2.917 ± 0.405	3.329 ± 0.502	0.001*
	TT4RI	36.768 ± 13.389	46.890 ± 19.859	0.031*
**Post-operative**	TSH (mU/L)	0.292 (0.139,0.688)	6.000 (6.195,9.992)	<0.001*
	FT3 (pmol/L)	4.669 ± 0.600	4.377 ± 0.552	0.062
	FT4 (pmol/L)	20.403 ± 1.286	24.698 ± 1.888	<0.001*
	FT3/FT4 ratio	0.230 ± 0.032	0.178 ± 0.025	<0.001*
	TSHI	1.421 (0.655,2.418)	5.289 (4.813,5.615)	<0.001*
	TT4RI	6.058 (2.764,14.28)	159.943 (121.094,255.609)	<0.001*
L-T4 dosage (ug/kg)		1.494 (1.3636,1.7054)	1.592 (1.506,1.718)	0.140

*p<0.05.

**Figure 2:**
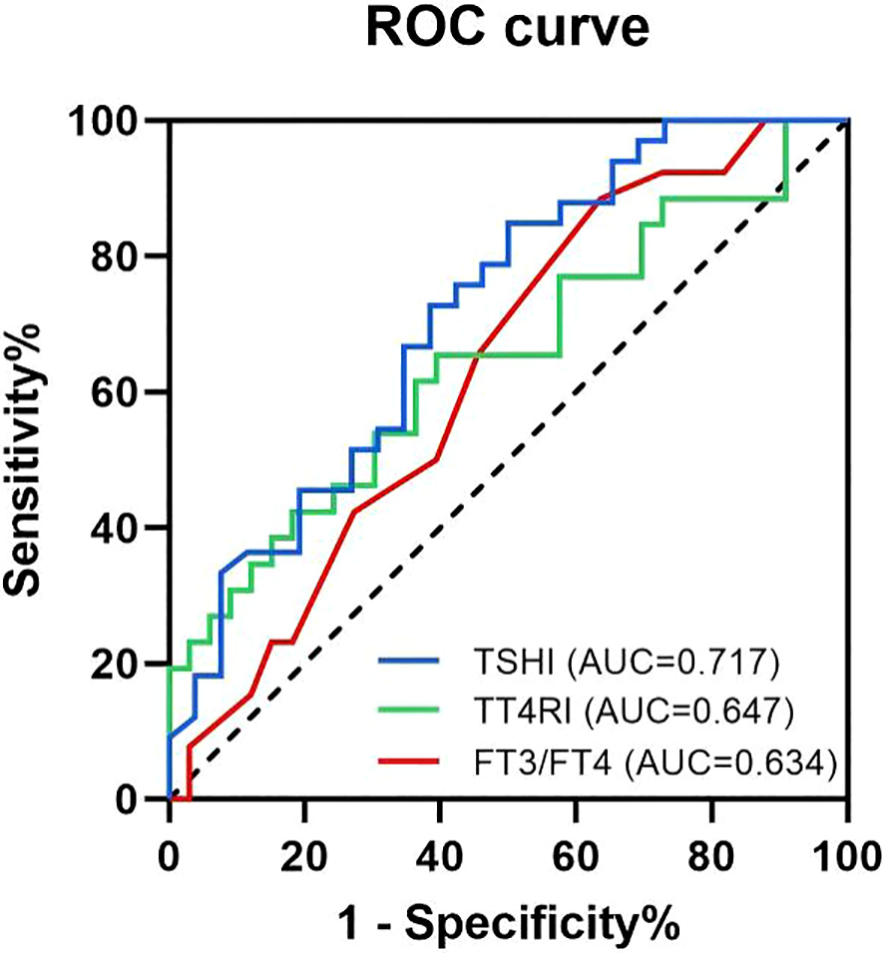
Receiver operating characteristic (ROC) curves and the area under the ROC curve for the predicting factors. The blue curve is the ROC curve for TSHI, with an AUC of 0.717 (95% CI: 0.584-0.849; p=0.005). The green curve is the ROC curve for TT4RI, with an AUC of 0.647 (95% CI: 0.503-0.791; p=0.054). The red curve is the ROC curve for FT3/FT4 ratio, with an AUC of 0.634 (95% CI: 0.492-0.776; p=0.072).

The value of TSHI, TT4RI and FT3/FT4 ratio in TSH suppressed subgroup and TSH not-suppressed subgroup were used to draw ROC curve, and AUC were determined (**Figure 2**). The AUC of TSHI was 0.717 (95% confidence interval (CI): 0.584–0.849; p = 0.005). The AUC of TT4RI was 0.647 (95% CI: 0.503-0.791; p = 0.054). The AUC of FT3/FT4 was 0.634 (95% CI: 0.492–0.776; p = 0.072). Further analysis shwed that the cut-off value of TSHI was 0.348 with a 84.8% specificity and 50.0% sensitivity for predicting situations of high postoperative TSH levels even when receiving sufficient amount of L-T4. Binary logistic regression confirmed TSHI as an independent predictor of blunted response to TSH suppression therapy post-total thyroidectomy, controlling for sex, age, I-131 therapy, and preoperative thyroid function parameters (TSH, FT3, FT4, ratios, TSHI, TT4RI) (**Table 4**).

**Table 4:** Binary logistic regression analysis of TSH non-suppression following TSH suppression therapy.

Variables	B	S.E.	*p*	Exp(B)	95% CI for EXP(B)	
					Lower	Upper
Gender	-0.56	0.366	0.126	0.571	0.279	1.17
Age	-0.025	0.015	0.111	0.976	0.946	1.006
I^131^	0.247	0.372	0.506	1.28	0.618	2.654
preoperative TSH	0.72	1.745	0.68	2.054	0.067	62.768
preoperative FT3	2.168	1.886	0.25	8.744	0.217	352.68
preoperative FT4	0.031	0.67	0.963	1.031	0.277	3.837
preoperative TSHI	-4.186	1.996	0.036*	0.015	0	0.76
preoperative TT4RI	0.076	0.094	0.422	1.079	0.897	1.297
Preoperative FT3/FT4 ratio	-29.744	32.476	0.36	0	0	5.320×10^14
Constant	6.648	11.163	0.551	771.584		

*P < 0.05.

**Figure 3:**
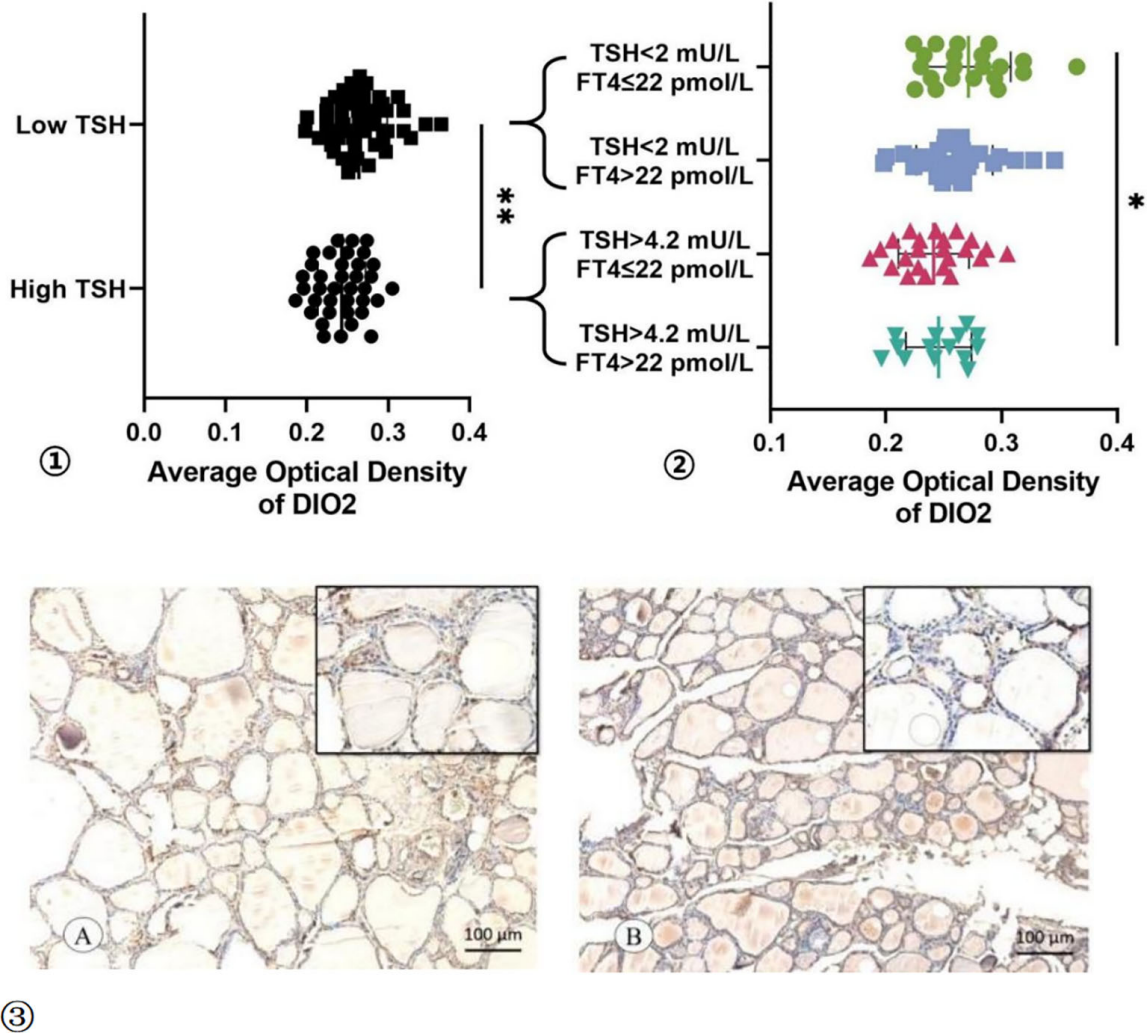
The AOD of DIO2 Expression. ① The AOD of DIO2 in Low TSH group and High TSH group. ② The AOD of DIO2 in different subgroup. ③ The immunohistochemistry results of the DIO2 expression in TSH suppressed subgroup **(A)** and TSH not-suppressed subgroup **(B)**. * p < 0.05, ** p < 0.01.

Then, to investigate the mechanisms why TSH cannot be suppressed to the desired range after undergoing thyroid cancer surgery. The protein expression levels of DIO2 were detected in normal thyroid tissue adjacent to thyroid cancer tissues using immunohistochemistry (**Figure 3**). The AOD of DIO2 was higher in the Low TSH group than High TSH group (0.264 ± 0.035 vs 0.243 ± 0.029; t = 3.018, p = 0.003). This difference remained between the TSH suppressed subgroup and TSH not-suppressed subgroup (0.271 ± 0.037 vs 0.246 ± 0.028; t = 2.222, p = 0.033). Among patients with normal FT4 levels, the expression of DIO2 was significantly higher in the Low TSH group compared to the High TSH group(0.271 ± 0.037 vs 0.241 ± 0.031; t = 2.971, p = 0.005).

## 4 Discussion

The level of TSH is closely related to cancer recurrence, metastasis and mortality in DTC patients. TSH suppression in patients with DTC should be optimized to reduce tumor recurrence while minimizing the risk of toxicity associated with subclinical hyperthyroidism (18). It is currently recognized that when TSH reaches more than 2 mU/L, the risk of thyroid cancer-related recurrence and death increases (19). A TSH level less than 2 mU/L could be used as the basic threshold to determine whether TSH suppression therapy achieved the target.This study focused on DTC patients undergoing TSH suppression therapy after total thyroidectomy. We evaluated their thyroid function and thyroid hormone sensitivity indices. Additionally, DIO2 expression in postoperative normal thyroid tissues was also analyzed. We found that in some patients, although postoperative FT4 levels increased and even exceeded the upper limit of the normal reference range, TSH could still not be suppressed to normal levels or the desired range.

Patients undergone total thyroidectomy often required higher levels of FT4 than before surgery to maintain stable thyroid function. More T4 to T3 conversion is needed to provide total T3 requirement and compensate for the lack of T3 synthesis. In our study, the postoperative FT4 levels in all patients were higher than the preoperative levels, indicating sufficient L-T4 dosages were used. During TSH suppression therapy, compared to patients who achieved the basic target of TSH suppression therapy, patients with high TSH levels had lower T3 levels. This hormonal pattern may enhance the negative feedback regulation of thyroid hormones on TSH secretion, requiring an increased dose of L-T4 during TSH suppression therapy to maintain TSH levels within the target range. In our study, the High TSH group received a higher average dose of L-T4 than did the low TSH group, and the TSH not-suppressed subgroup received a higher average dose of L-T4 than did the TSH suppressed subgroup. It is obvious that a sufficient and even supraphysiological dose of L-T4 did not suppress the TSH to the desired level in these patients. For these patients, L-T4 monotherapy may provide incomplete therapeutic effects to suppress TSH levels to the target range. A combination therapy with T3-containing medications such as liothyronine or desiccated thyroid extract should be considered (20, 21).

Total thyroidectomy eliminates endogenous T3 production, rendering peripheral T4-to-T3 conversion via deiodinases (DIO1/DIO2) the exclusive source of T3. Both DIO1 and DIO2 are able to convert T4 to T3 but DIO2 playing a much more efficient role in this conversion (12, 22). DIO1 shows 1000-fold lower T4 affinity than DIO2 (23). While type III deiodinase (DIO3) inactivates both T4 and T3. The biological activity of T3 is much higher than T4 (24). Therefore, we speculate that postoperative patients with high TSH levels may have an inherent deiodinases dysfunction. Such dysfunction may result in impaired T4-to-T3 conversion. Elevated DIO2 activity is predominantly observed in the brain, pituitary gland, and brown adipose tissue under cold stimulation (25); it also expressed in skin, skeletal muscle, bone, vascular smooth muscle, and testes (23). DIO1 is highly expressed in the thyroid, liver, and kidneys (25).

The HPT axis and the synergistic action of deiodinases are crucial for maintaining thyroid homeostasis. TSH-induced thyroidal T3 secretion is the gateway through which the HPT axis controls systemic thyroid hormone signaling (26).The inhibition of TSH secretion is predominantly facilitated by T4 and its intracellular conversion to T3 (27). In pituitary, the expression of DIO2 is abundant, establishing an intracellular T3 gradient (3–5 times higher than serum concentrations) that drives TSH negative feedback regulation (12). In pituitary thyrotrophs, T3-TRβ2 binding suppresses TSHβ and thyrotropin-releasing hormone receptor gene transcription (12, 25). Concurrently, paracrine T4 modulates DIO2 stability via ubiquitin-proteasome pathways (28). Together, these processes coordinate thyroid hormone feedback regulation. Reciprocally, the HPT axis modulates DIO2 activity via dual mechanisms: (1) TSH-stimulated upregulation of DIO2 expression in thyroid cells amplifies peripheral T3 production, and (2) elevated thyroid hormones induce DIO2 degradation through ubiquitination (29, 30). This self-tuning circuit ensures precise control of thyroid hormone homeostasis through balanced feedforward and feedback mechanisms.

However, direct assessment of DIO expression in pituitary tissues was ethically prohibited. Because for patients with normal pituitary function, pituitary tissue puncture or biopsy may lead to serious complications. Future investigations will involve the collection of pituitary tissues from established animal models for systematic deiodinase profiling and characterization. This study aimed to elucidate the reasons for unsatisfactory TSH suppression. We conducted immunohistochemical analysis using postoperative specimens. The analysis specifically examined histologically normal thyroid tissue adjacent to tumor lesions. These specimens were obtained during total thyroidectomy procedures to evaluate systemic DIO2 expression patterns.

Our study found that the expression of DIO2 in normal thyroid tissue in patients with high TSH levels during levothyroxine (L-T4) treatment was significantly lower than those who achieved the target TSH level. Previous studies have shown that different thyroid function regulate DIO1 and DIO2 expression in the opposite manner: hyperthyroidism increases DIO1 and decreases DIO2 expression, while hypothyroidism results in the opposite effect (25). All enrolled patients had normal thyroid function before surgery in this study. Therefore, the expression of DIO2 was not affected by thyroid dysfunction preoperatively. To avoid the impact of abnormal postoperative FT4 levels on the expression of DIO2, we further analyzed the expression of DIO2 in the thyroid tissue of patients with normal FT4 levels when receiving TSH suppression therapy. It also showed that for patients with FT4 within the normal range, a higher TSH associates with a lower expression of DIO2 in the thyroid tissues.

Therefore, we speculate that, compared to those who achieved target levels in TSH suppression therapy, the expression of DIO2 is lower in patients with high TSH, not only in the thyroid but also in the pituitary. A lower DIO2 expression in the pituitary may lead to local T3 deficiency and increased secretion of TSH. Furthermore, genetic polymorphisms in the DIO2 gene, particularly the rs225014 (Thr92Ala) single-nucleotide polymorphism (SNP), were found to be associated with altered plasma thyroid hormone levels (12, 31, 32). This specific variant may directly reduce DIO2 enzymatic activity. Primary culturing of pituitary cells demonstrated an impairment in the T4 to T3 conversion by the mutant enzyme (31). Therefore, the actual expression and biological activity of DIO2 in the pituitary gland require further investigation. This is particularly important for patients demonstrating poor response to TSH suppression therapy.

Given the complex interactions within the HPT axis, indices such as TSHI and TT4RI were proposed to evaluate central thyroid hormone sensitivity (33). Our study noticed that before surgery, compared to the TSH suppressed subgroup, the TSH not-suppressed subgroup had significantly worse central thyroid hormone sensitivity (higher TSHI and TT4RI). After surgery, this difference between the two subgroups became even more pronounced.The serum FT3/FT4 ratio is used to evaluate the peripheral thyroid hormone sensitivity. Preoperatively, the TSH non-suppressed subgroup demonstrated slightly reduced peripheral thyroid hormone sensitivity compared to the TSH suppressed subgroup. Postoperatively, this difference became more pronounced. The TSH non-suppressed subgroup showed significantly worse peripheral thyroid hormone sensitivity after surgery. These results may indicate that individuals with poor response to TSH suppression therapy inherently have poorer central and peripheral thyroid hormones sensitivity, and total thyroidectomy will further worsen the situation.

The predictive capacity of each thyroid hormone sensitivity indice was assessed by ROC curve and the AUC. We found that among the three thyroid sensitivity indicators analyzed, TSHI demonstrated the strongest predictive ability. Patients with TSHI above 3.276 may have higher postoperative TSH levels even when they receive an sufficient dose of L-T4 treatment. The binary logistic regression results further corroborate these findings. Although their clinical utility is limited, they provide clinicians with diagnostic and therapeutic insights.

In conclusion, this study reveals that a subset of total thyroidectomy patients maintained elevated TSH levels during suppression therapy despite adequate L-T4 dosing. This phenomenon appears mechanistically linked to systematically reduced DIO2 expression, which compromises peripheral T4-to-T3 conversion. This was related to decreased FT3 levels, elevated FT4 levels and mismatched high TSH levels. Individuals with poor response to TSH suppression therapy inherently have poorer central and peripheral thyroid hormones sensitivity, and total thyroidectomy will further worsen the situation. In addition, elevated TSHI levels help to predict poor therapeutic outcomes in TSH suppression therapy when L-T4 is used as monotherapy. Our study provides new insights into the impact of thyroid hormone sensitivity on the efficacy of TSH suppression therapy and offers clinical evidence for elucidating the role of DIO2 in this process. Further research is needed to elucidate both the pituitary DIO2 expression profile and its functional role in the mechanism underlying unsatisfactory TSH suppression therapy.

## Data Availability

The raw data supporting the conclusions of this article will be made available by the authors, without undue reservation.
